# Identifying individual, household and environmental risk factors for malaria infection on Bioko Island to inform interventions

**DOI:** 10.1186/s12936-023-04504-7

**Published:** 2023-03-01

**Authors:** Guillermo A. García, Mark Janko, Dianna E. B. Hergott, Olivier T. Donfack, Jordan M. Smith, Jeremías Nzamío Mba Eyono, Kylie R. DeBoer, Restituto Mba Nguema Avue, Wonder P. Phiri, Edward M. Aldrich, Christopher Schwabe, Thomas C. Stabler, Matilde Riloha Rivas, Ewan Cameron, Carlos A. Guerra, Jackie Cook, Immo Kleinschmidt, John Bradley

**Affiliations:** 1 MCD Global Health, Silver Spring, MD USA; 2 Duke Global Health Institute, Duke University, Durham, NC USA; 3 Department of Epidemiology, School of Public Health, University of Washington, Seattle, WA USA; 4 MCD Global Health, Bioko Island, Malabo, Equatorial Guinea; 5 MCD Global Health, Hallowell, ME USA; 6 Department of Medical Parasitology and Infection Biology, Swiss Tropical and Public Health Institute, Basel, Switzerland; 7 University of Basel, Basel, Switzerland; 8 Equatorial Guinea Ministry of Health and Social Welfare, Bioko Island, Malabo, Equatorial Guinea; 9 Telethon Kids Institute, Perth Children’s Hospital, Perth, Australia; 10 MRC International Statistics and Epidemiology Group, London School of Hygiene and Tropical Medicine, London, UK; 11 School of Pathology, Faculty of Health Science, Wits Institute for Malaria Research, University of Witwatersrand, Johannesburg, South Africa

**Keywords:** Malaria, Targeted interventions, Importation, Vector control, Risk factors

## Abstract

**Background:**

Since 2004, malaria transmission on Bioko Island has declined significantly as a result of the scaling-up of control interventions. The aim of eliminating malaria from the Island remains elusive, however, underscoring the need to adapt control to the local context. Understanding the factors driving the risk of malaria infection is critical to inform optimal suits of interventions in this adaptive approach.

**Methods:**

This study used individual and household-level data from the 2015 and 2018 annual malaria indicator surveys on Bioko Island, as well as remotely-sensed environmental data in multilevel logistic regression models to quantify the odds of malaria infection. The analyses were stratified by urban and rural settings and by survey year.

**Results:**

Malaria prevalence was higher in 10–14-year-old children and similar between female and male individuals. After adjusting for demographic factors and other covariates, many of the variables investigated showed no significant association with malaria infection. The factor most strongly associated was history of travel to mainland Equatorial Guinea (mEG), which increased the odds significantly both in urban and rural settings (people who travelled had 4 times the odds of infection). Sleeping under a long-lasting insecticidal net decreased significantly the odds of malaria across urban and rural settings and survey years (net users had around 30% less odds of infection), highlighting their contribution to malaria control on the Island. Improved housing conditions indicated some protection, though this was not consistent across settings and survey year.

**Conclusions:**

Malaria risk on Bioko Island is heterogeneous and determined by a combination of factors interacting with local mosquito ecology. These interactions grant further investigation in order to better adapt control according to need. The single most important risk factor identified was travel to mEG, in line with previous investigations, and represents a great challenge for the success of malaria control on the Island.

**Supplementary Information:**

The online version contains supplementary material available at 10.1186/s12936-023-04504-7.

## Background

**Fig. 1:**
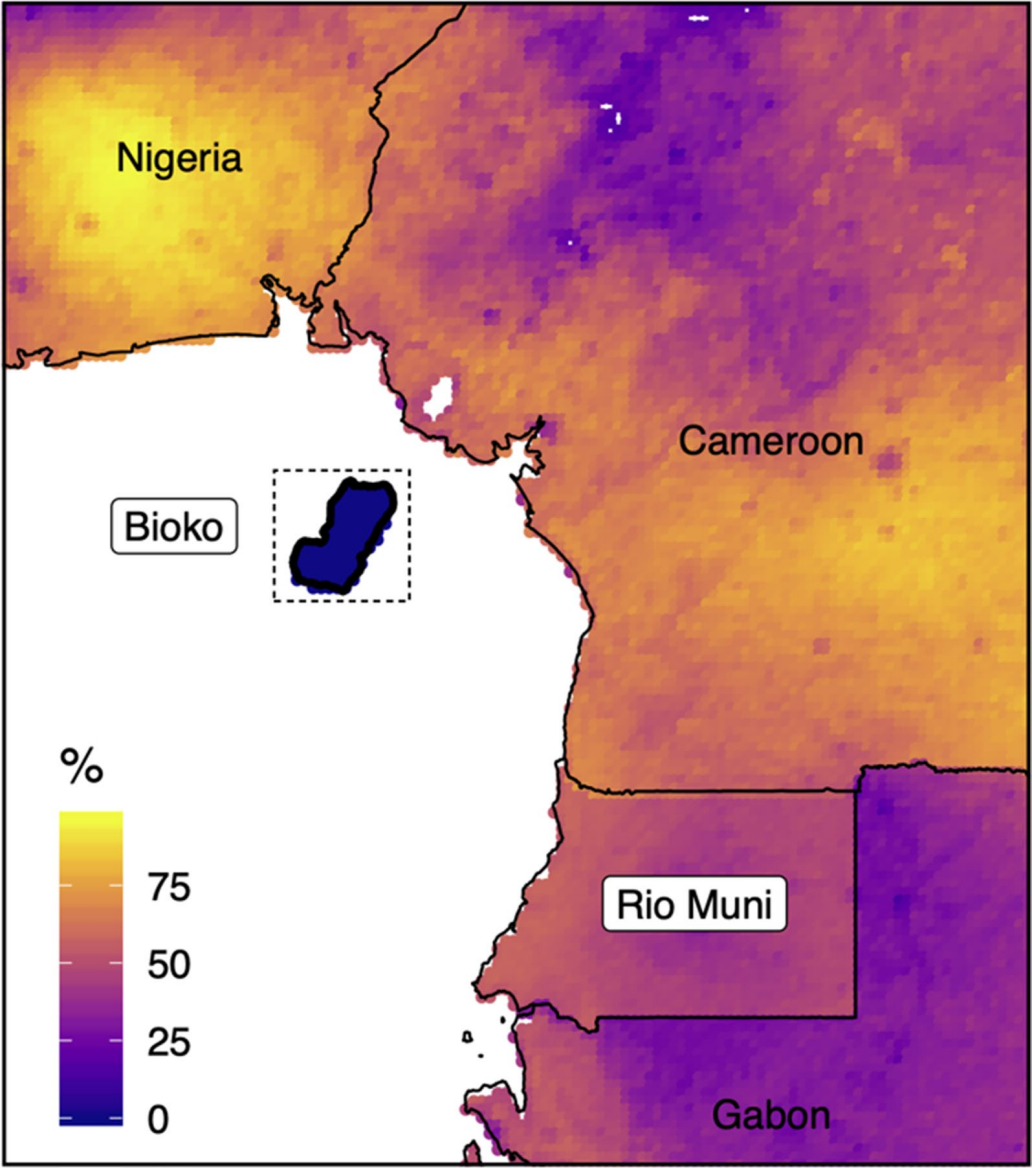
Bioko Island and its location in the Gulf of Guinea. The continental territory of Equatorial Guinea is known as Río Muni. The color scale represents predicted *Pf*PR in children, reconstructed from data produced by the Malaria Atlas Project, which are available for use under the Creative Commons Attribution 3.0 Unported License

Bioko is the largest Island of Equatorial Guinea (EG), with an area of 2,017 km2. It is located in the Bight of Biafra, off the coast of Cameroon and has an approximate population of around 270,000 people [1]. (Fig. 1). Most of the population lives on the north side of the Island, near and within Malabo, the country capital. Historically, Bioko was hyperendemic for malaria transmission, with some of the highest entomological inoculation rates (EIR) ever recorded: over one thousand infective bites per person per year, 281 for *Anopheles gambiae *sensu lato (*s.l*.) and 787 for *Anopheles funestus* [2–4] This translated into a pre-intervention *Plasmodium falciparum* parasite rate (PfPR) of 45% in 2–14-year-old children [5].

**Fig. 2:**
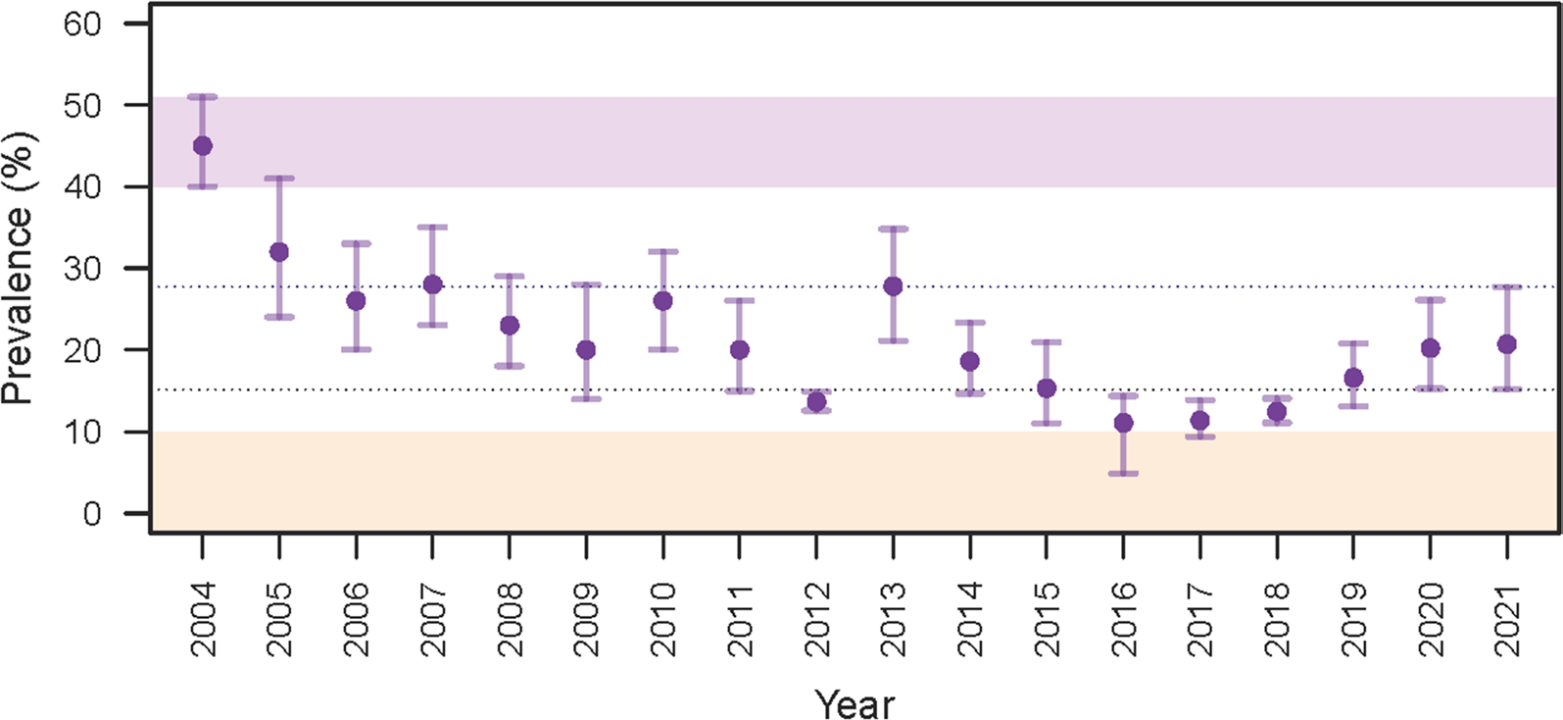
Malaria prevalence in children 2–14 years old on Bioko Island, 2004–2021

In 2004, the Bioko Island Malaria Elimination Project (BIMEP) was established and has since successfully reduced the malaria burden [6, 7]. PfPR in 2–14-year-old children has declined by about 75%, and all-cause mortality and anaemia in under 5-year-olds by 63% and 86%. Another critical benchmark of the project was the elimination of two of the principal vectors, *An. funestus* and *An. gambiae *sensu stricto, from the Island [2–4], leaving only An. coluzzii and An. Melas [8], which sustain an EIR in order of magnitude lower than in pre-intervention times. PfPR in the lowest year on record (2016) was between 10 and 15% across the Island (Fig. 2), substantially lower than in the surrounding neighbours (Fig. 1). Despite these gains, several areas on the Island have seen a resurgence of malaria similar to other endemic regions across Africa (Fig. 2) [9].

At the inception of the BIMEP, whole-island indoor residual spraying (IRS) was the primary vector control strategy supplemented by limited long-lasting insecticidal net (LLIN) distributions. In the early stages of the Project, it was believed that IRS could rid Bioko of malaria in a few years, but this was soon realized as an overly optimistic goal and this led to attempts to adapt strategies in order to sustain gains. Starting in 2015, two triennial LLIN mass distribution campaigns (MDC) were conducted, one in 2015 and one in 2018, which came to represent the main vector control intervention. LLINs were supported by targeted IRS in high prevalence areas together with intermittent preventive treatment for pregnant women, case management using artemisinin-based combination therapy (ACT), diagnosis through training in microscopy and Rapid Diagnostic Tests (RDTs), and larval source management [6, 10].

A lingering question is whether targeting interventions or universal coverage of the population is the best approach to control malaria while making optimal use of limited resources [11]. Targeting strategies have used statistical models to identify malaria hotspots based on malaria prevalence and malaria incidence data, demographic factors, and transmission etiology [12–19]. Targeting of IRS has proven particularly unsuccessful on Bioko, however, as malaria hotspots represent moving spatial targets around the Island. Therefore, a comprehensive and regularly updated understanding of intervention effectiveness and risk factors for malaria and how they may vary by context, and over time, would prove critical to any targeting strategy. Here, the study evaluated individual, household, and environmental risk factors on Bioko to improve the understanding of some of the main drivers of malaria transmission on the Island.

## Methods

### MIS Data

The study used individual and household-level data from the 2015 and 2018 annual malaria indicator surveys (MIS). The sampling frame for the MIS was drawn using a comprehensive database that uniquely identifies and geo-references all households on Bioko Island [20]. Clusters were defined by single communities containing at least 20 households and, in the case of sparsely populated communities, an aggregation of communities. A random sample of households was then selected from these clusters comprising 7% of the total island household universe in 2015 and 6% in 2018. Consenting household members present during the survey were tested for *P. falciparum* using RDTs (CareStart G0131 Combo kit, AccessBio Inc., Monmouth, USA). In the case of children, consent was sought from their guardians.

The data on individuals, households, and covariates collected during the MIS and explored in this study are listed in Table 1. Individual-level data used in the analyses included age, sex, history of recent travel to mainland EG (mEG), and LLIN use the previous night. Household-level data included socioeconomic status (SES), household density, and housing characteristics. To derive SES, households were assigned scores based on the type of assets and amenities they own (radio, television, sofa, fan, air-conditioning, car, among others) using principal component analysis (PCA). Households were ranked based on their score and divided into quintiles. The first quintile corresponded to the lowest wealth index (WI) and the fifth to the highest WI. Household density was defined as the ratio of the number of household occupants to the number of rooms in the house and was classified into five categories: very low (≤ 0.5), low (> 0.5–  < 1), medium (≥ 1– < 1.5), high (≥ 1.5– < 2) and very high (≥ 2). Housing characteristics included whether the house had open eaves or air conditioning in place and distance to specific geographical features of interest, namely bodies of water and military camps. Military camps may be important in this context because military personnel represent a source of human infectivity due to frequent travel to mEG [21].

**Table 1:** Individual, Household, and Environmental covariates investigated in the study

Individual	Source	Included in Model
Age	Malaria Indicator Survey	x
Gender		x
Household size		x
Household density		x
Travel History		x
Socioeconomic Status		x
LLIN use		x
Household		
Wall Type	Malaria Indicator Survey	
Wall Gaps		
Roof Type		
Roof Types		
Floor Types		
Air Conditioner ownership		x
Window Glass		
Window Screen		
Door Screen		
Eaves		x
Water Source		
Light Source		
Toilet Type		
Environmental		
Elevation	Shuttle Radar Topography Mission	x
Slope		x
Size of community	BIMEP Mapping System	
Distance to water streams		x
Distance to military camps		x
Population density		
Tasseled Cap Brightness (TCB)	Nasa Earth Data	x
Tasseled Cap Wetness (TCW)		x
Land Surface Temperature (LST)		x
Enhanced Vegetation Index (EVI)		x

### Environmental data

Remote sensing-derived environmental data included the Tasseled Cap Brightness (TCB), the Tasseled Cap Wetness (TCW) [22], Land Surface Temperature (LST) [22], the Enhanced Vegetation Index (EVI) [23], and elevation and slope derived from the Shuttle Radar Topography Mission [24]. Information on how TCB, TCW, LST and EVI are compiled and processed is described elsewhere [7, 25].

### Statistical analysis

The primary outcome of interest was individual-level *P. falciparum* malaria infection, as determined by the RDT result reported in the MIS. The covariates listed in Table 1 were investigated for their association with malaria infection using multilevel logistic regression, controlling for potential confounders. To account for the survey design, a random intercept from the MIS survey cluster was included and modeled for its random effect. To account for variability due to differing operational strategies deployed in urban and rural settings, the models were stratified to observe their associations separately. Finally, because the study aimed at identifying the persistence of risk factors across time, the analysis was also stratified by survey year (2015 vs. 2018). All continuous covariates were centered and scaled such that inferences were based on a one standard deviation change in the covariate of interest. All statistical analyses were run in Stata version 16 [26].

## Results

### Descriptive statistics

In the 2015 and 2018 MIS, 17,016 and 13,906 individuals were tested for malaria. However, some of these individuals had incomplete survey information and were excluded from the analyses. As a result, 16,903 individuals living in 5184 households surveyed in 2015 and 13,734 individuals living in 4762 households surveyed in 2018 were included. Overall, 55.2% of surveyed individuals were female and 84.6% lived in urban areas. The median age of survey participants was 14 years. Roughly a quarter of individuals lived in each of the SES quintiles, with the most in the poorest (26.5%) and the least in the wealthiest (23.4%). Most people lived in medium density households (38.7%), though almost 8% lived in very high density households. The majority of individuals lived in households with open eaves (68.0%) and no air conditioning (81.6%). Around half reported sleeping under a net the night before the survey (55.1%) and 9.5% reported travelling to mEG within 2 months prior to the survey. Descriptive statistics of these variables by each MIS survey and urban/rural stratum are presented in Table 2.

**Table 2:** Descriptive statistics for individual, household, and environmental covariates included in regression analyses

Covariate	2015			2018			Total 2015 and 2018		
	Urban	Rural	All	Urban	Rural	All	Urban	Rural	All
	(n = 14,476)	(n = 2427)	16,903	(n = 11,444)	(n = 2290)	13,734	(n = 25,920)	(n = 4,717)	(n = 30,637)
Sex									
Male	6295 (43.5)	1101 (45.4)	7396 (45.4)	5167 (45.2)	1152 (50.3)	6319 (46.0)	11,462 (44.22)	2253 (47.76)	13,715 (44.77)
Female	8181 (56.5)	1326 (54.6)	9507 (54.6)	6277 (54.8)	1138 (49.7)	7415 (54.0)	14,458 (55.78)	2464 (52.24)	16,922 (55.23)
Age group									
< 5 years	3086 (21.3)	470 (19.4)	3556 (21.0)	1646 (14.4)	285 (12.5)	1931 (14.1)	4732 (18.3)	755 (16.0)	5487 (17.9)
5–9 years	1909 (13.2)	329 (13.6)	2238 (13.2)	1450 (12.7)	279 (12.2)	1729 (12.6)	3359 (13.0)	608 (12.9)	3967 (12.9)
10–14 years	1781 (12.3)	276 (11.4)	2057 (12.2)	1421 (12.4)	274 (12.0)	1695 (12.3)	3202 (12.4)	550 (11.7)	3752 (12.2)
15–19 years	1422 (9.8)	160 (6.6)	1582 (9.4)	1127 (9.8)	200 (8.7)	1327 (9.7)	2549 (9.8)	360 (7.6)	2909 (9.5)
20–29 years	3101 (21.4)	374 (15.4)	3475 (20.6)	2378 (20.8)	394 (17.2)	2772 (20.2)	5479 (21.1)	768 (16.3)	6247 (20.4)
30–39 years	1580 (10.9)	253 (10.4)	1833 (10.8)	1534 (13.4)	278 (12.1)	1812 (13.2)	3114 (12.0)	531 (11.3)	3645 (11.9)
40–49 years	731 (5.1)	219 (9.0)	950 (5.6)	642 (5.6)	154 (6.7)	796 (5.8)	1373 (5.3)	373 (7.9)	1746 (5.7)
50–59 years	480 (3.3)	166 (6.8)	646 (3.8)	419 (3.7)	163 (7.1)	582 (4.2)	899 (3.5)	329 (7.0)	1228 (4.0)
> 60 years	386 (2.7)	180 (7.4)	566 (3.4)	827 (7.2)	263 (11.5)	1090 (7.9)	1213 (4.7)	443 (9.4)	1656 (5.4)
Socioeconomic Status (Quantile)									
Poorest	3036 (21.0)	1443 (59.5)	4479 (26.5)	2616 (22.9)	1,022 (44.6)	3638 (26.5)	5652 (21.8)	2465 (52.3)	8117 (26.5)
2nd	3812 (26.3)	532 (21.9)	4344 (25.7)	2911 (25.4)	527 (23.0)	3438 (25.0)	6723 (25.9)	1059 (22.5)	7782 (25.4)
3rd	3840 (26.5)	281 (11.6)	4121 (24.4)	2980 (26.0)	454 (19.8)	3434 (25.0)	6820 (26.3)	735 (15.6)	7555 (24.7)
Wealthiest	3788 (26.2)	171 (7.0)	3959 (23.4)	2937 (25.7)	287 (12.5)	3224 (23.5)	6725 (25.9)	458 (9.7)	7183 (23.4)
Household density									
Very low density	1585 (11.0)	483 (19.9)	2068 (12.2)	1388 (12.1)	448 (19.6)	1836 (13.4)	2973 (11.5)	931 (19.7)	3904 (12.7)
Low density	4217 (29.1)	684 (28.2)	4901 (29.0)	2966 (25.9)	580 (25.3)	3546 (25.8)	7183 (27.7)	1264 (26.8)	8447 (27.6)
Medium density	6104 (42.2)	708 (29.2)	6812 (40.3)	4341 (37.9)	712 (31.1)	5053 (36.8)	10,445 (40.3)	1420 (30.1)	11,865 (38.7)
High density	1802 (12.4)	351 (14.4)	2153 (12.7)	1582 (13.8)	234 (10.2)	1816 (13.2)	3384 (13.1)	585 (12.4)	3969 (12.9)
Very high density	768 (5.3)	201 (8.3)	969 (5.7)	1167 (10.2)	316 (13.8)	1483 (10.8)	1935 (7.5)	517 (11.0)	2452 (8.0)
LLIN Use									
Yes	7681 (53.1)	1312 (54.1)	8993 (53.2)	6633 (58.0)	1251 (54.6)	7884 (57.4)	14,314 (55.2)	2563 (54.3)	16,877 (55.1)
No	6795 (46.9)	1115 (45.9)	7910 (46.8)	4811 (42.0)	1039 (45.4)	5850 (42.6)	11,606 (44.8)	2154 (45.7)	13,760 (44.9)
Travelled to continental Africa									
Yes	1933 (13.4)	134 (5.5)	2067 (12.2)	773 (6.8)	76 (3.3)	849 (6.2)	2706 (10.4)	210 (4.5)	2916 (9.5)
No	12,543 (86.6)	2293 (94.5)	14,836 (87.8)	10,671 (93.2)	2214 (96.7)	12,885 (93.8)	23,214 (89.6)	4507 (95.5)	27,721 (90.55)
House has open eaves									
Yes	10,592 (73.2)	1191 (49.1)	11,783 (69.7)	7712 (67.4)	1345 (58.7)	9057 (66.0)	18,304 (70.6)	2536 (53.8)	20,840 (68.0)
No	3884 (26.8)	1236 (50.9)	5120 (30.3)	3732 (32.6)	945 (41.3)	4677 (34.0)	7616 (29.4)	2181 (46.2)	9797 (32.0)
House has air conditioning									
Yes	2228 (15.4)	167 (6.8)	2395 (14.2)	2819 (24.6)	408 (17.8)	3227 (23.5)	5047 (19.5)	575 (12.2)	5622 (18.4)
No	12,244 (84.6)	2260 (93.1)	14,504 (85.8)	8625 (75.4)	1882 (82.2)	10,507 (76.5)	20,869 (80.5)	4142 (87.8)	25,011 (81.6)
Environmental covariates, mean (sd)									
TCB	0.52 (0.08)	0.5 (0.14)	0.52 (0.09)	0.37 (0.03)	0.38 (0.03)	0.37 (0.03)	N/a	N/a	N/a
TCW	− 0.01 (0.02)	0.03 (0.04)	0.00 (0.03)	− 0.02 (0.02)	0 (0.03)	− 0.02 (0.02)	N/a	N/a	N/a
Elevation (meters)	71.67 (77.62)	284.09 (362.75)	102.98 (172.48)	70.54 (70.21)	299.54 (384.02)	110 (190.05)	N/a	N/a	N/a
Temperature (C)	22.81 (3.61)	23.43 (4.43)	22.88 (3.78)	23.43 (2.12)	23.06 (2.67)	23.36 (2.23)	N/a	N/a	N/a
EVI	0.24 (0.11)	0.46 (0.11)	0.27 (0.14)	0.25 (0.13)	0.49 (0.12)	0.29 (0.16)	N/a	N/a	N/a
Slope	7.61 (4.15)	10.13 (6.33)	7.97 (4.61)	7.5 (4.07)	10.37 (6.30)	7.99 (4.65)	N/a	N/a	N/a

### Malaria prevalence

Prevalence of *P. falciparum* infection was 12.7% (CI 11.4–14.2, p < 0.05) in 2015 and 10.2% (CI 9.3–11.3, p < 0.05) in 2018. This translated into 25.5% and 20.1% of surveyed households in 2015 and 2018, respectively, having at least one *P. falciparum-*positive household member. Table 3 shows the prevalence of malaria infection by risk factor and survey year. In 2015 and 2018, malaria prevalence was highest in the 10–14 years age group at 21.8% and 16.5%, respectively. Women had a lower prevalence than men in both years (11.9% vs. 13.8% in 2015 and 9.7% vs. 10.9% in 2018, respectively). In 2015 malaria prevalence was significantly higher in rural (17.0%) compared to urban areas (12.0%), though no significant difference was observed in 2018 (10.9% in urban vs 10.1% in rural areas). People living in very low-density households (8.6% in 2015 and 7.7% in 2018) had significantly lower prevalence than those living in very high-density households (15.9% in 2015 and 15.7% in 2018). People with a history of travel to continental EG had more than double the prevalence of those who did not travel (28.7% vs. 10.9% in 2015 and 30.4% vs. 9.2% in 2018).

**Table 3:** Prevalence of malaria infection by risk factor and malaria indicator survey year

	2015			2018		
	Malaria Prevalence %	CI	p-value	Malaria Prevalence %	CI	p-value
Sex						
Male	13.8	12.3–15.3	0.0005	10.9	9.8–12.1	0.0109
Female	11.9	10.5–13.5		9.7	8.6–10.8	
Age group						
< 5 years of age	8.8	7.5–10.3	< 0.0001	7.8	6.5–9.5	< 0.0001
5–9 years	17.8	15.3–20.6		13.5	11.7–15.5	
10–14 years	21.8	19.0–24.9		16.5	14.3–18.9	
15–19 years	18.3	16.1–20.8		14.8	12.9–17.3	
20–29 years	11.2	10.0–12.7		9.4	8.2–10.8	
30–39 years	8.3	6.9–9.9		8.2	7.0–9.8	
40–49 years	8.5	6.7–10.8		8.0	6.3–10.2	
50–59 years	7.7	5.6–10.7		5.4	3.8–8.1	
> 60 years of age	5.3	3.5–7.9		3.4	2.4–4.8	
Community location						
Urban	12.0	10.6–13.6	0.0090	10.9	8.3–14.3	0.6014
Rural	17.0	13.5–21.3		10.1	9.1–11.2	
Socioeconomic Status						
Poorest	13.6	11.3–16.1	0.0001	10.4	8.9–12.0	0.1809
2nd	13.6	11.8–15.5		10.8	9.5–12.4	
3rd	14.2	12.4–16.3		10.8	9.3–12.5	
Wealthiest	9.4	8.1–10.8		8.9	7.5–10.6	
Household density						
Very low density	8.6	7.1–10.4	< 0.0001	7.7	6.6–9.1	< 0.0001
Low density	10.5	8.8–12.4		8.5	7.2–9.9	
Medium density	13.8	12.2–15.5		10.3	9.1–11.6	
High density	17.1	14.8–19.7		11.6	9.2–14.5	
Very high density	15.9	12.6–19.9		15.7	13.1–18.7	
LLIN use						
Yes	10.8	9.5–12.2	< 0.0001	8.3	7.4–9.4	< 0.0001
No	14.9	13.2–16.8		12.8	11.4–14.3	
Travel to mainland, EG						
Yes	28.7	26.0–31.6	< 0.0001	30.4	25.7–35.6	< 0.0001
No	10.9	9.6–12.4		9.2	8.4–10.2	
House has open eaves						
Yes	11.9	10.7–13.3	0.0084	10.9	9.4–12.5	0.2758
No	14.6	12.4–17.2		9.9	8.9–11.1	
House has air conditioning						
Yes	9.2	7.5–11.3	0.0008	10.3	8.6–12.3	0.9576
No	13.3	11.9–14.9		10.4	9.2–11.3	
Environmental covariates	Mean (sd)			Mean (sd)		
TCB	0.52 (0.09)			0.37 (0.03)		
TCW	0.00 (0.03)			− 0.02 (0.02)		
Elevation (meters)	103 (172.49)			110 (190)		
Temperature (C)	22.88 (3.78)			23.36 (2.23)		
EVI	0.27 (0.14)			0.29 (0.16)		
Slope	7.97 (4.61)			7.99 (4.65)		

### Odds of malaria infection

#### Individual level factors

**Fig. 3:**
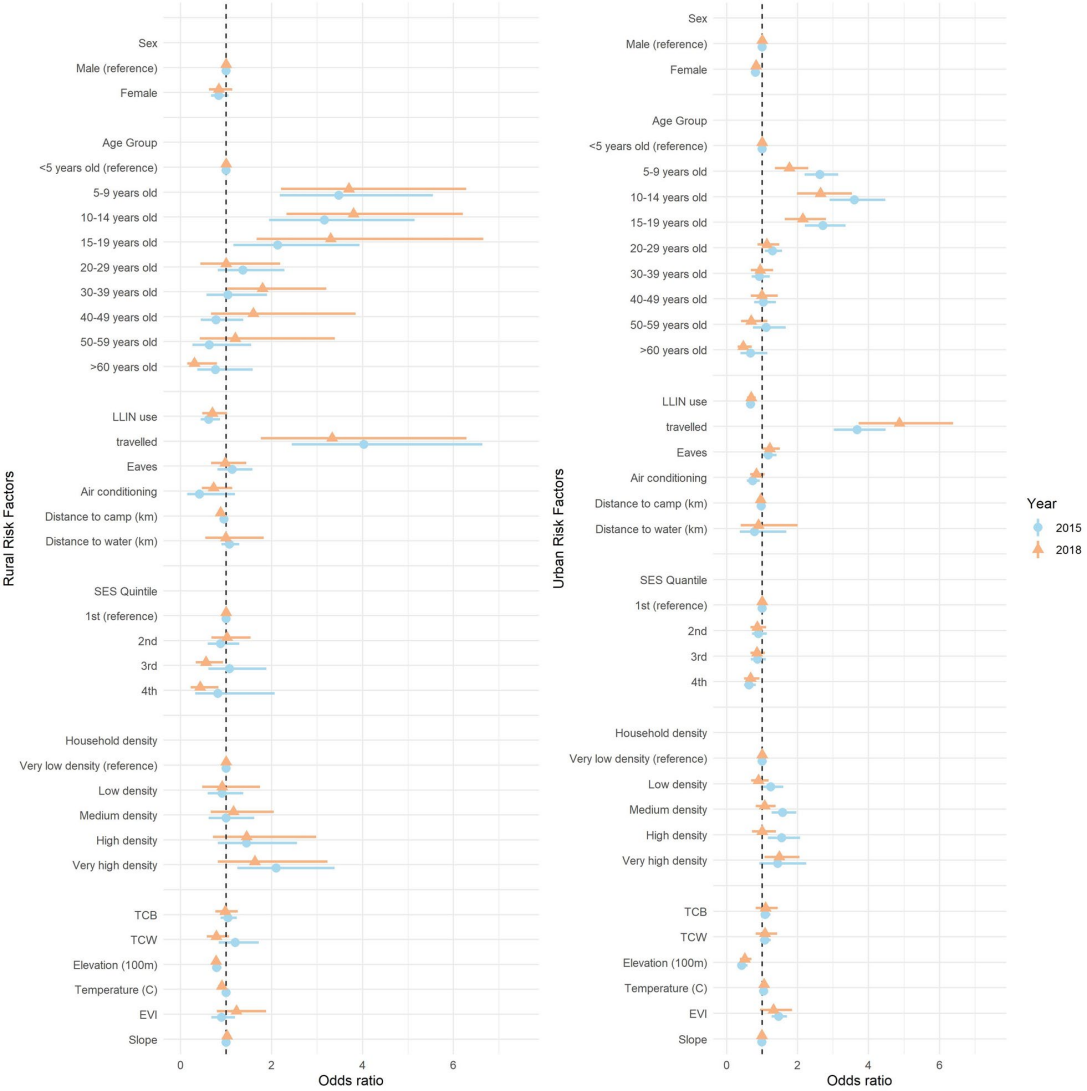
Odds ratios estimated from regression models of all risk factors for malaria transmission on Bioko island and 95% confidence intervals, stratified by rural (left) and urban (right) settings and malaria annual indicator survey year, 2015 (blue) and 2018 (orange)

An association between female sex and lower odds of infection was detected in urban settings but this was not significant in rural settings. The odds of infection in 2–14-year-old children were consistently and significantly higher. In both settings, individuals who slept under an LLIN the previous night had lower infection odds than those who did not (OR 0.67, 95% CI 0.48–0.93, *p* < 0.05 and OR 0.70, 95% CI 0.48–1.01, p = 0.06 in 2015 in rural areas, and OR 0.66, 95% CI 0.58–0.75 in 2015 and OR 0.70, 95% CI 0.59–0.83, p < 0.05 in 2018 in urban areas). In both years travel to mainland Equatorial Guinea was identified as the risk factor most strongly associated with the odds of malaria infection irrespective of setting, with those who travelled showing three to almost five times as likely to be infected than those who did not (OR 3.54, 95% CI 2.92–4.30, p < 0.05 in 2015 and OR 4.78, 95% CI 3.70–6.17, p < 0.05 in 2018 in urban areas, and OR 3.69, 95% CI 2.34–5.82, p < 0.05 in 2015 and OR 3.28, 95% CI 1.82–5.90, p < 0.05 in 2018 in rural areas; Fig. 3, Additional file 1: Table S1).

#### Household level factors

At the household level, multivariate regression identified no significant effect for distance to water bodies. Except for rural settings in 2015, wealth had significant protective effects against malaria infection, with individuals living in the wealthiest households having between a third and a half lower the odds of being infected (*p* < 0.05), with respect to those in the poorest households (Additional file 1: Table S1). Very high household density was a significant risk factor for those living in the densest households relative to those in the least dense ones, but only in rural areas in 2015 (OR 1.93, 95% CI 1.21–3.10, *p* < 0.05) and in urban areas in 2018 (OR 1.50, 95% CI 1.11–2.03, *p* < 0.05). A similar inconsistency was found in the effect on individuals living furthest away from military camps, who had significantly lower odds of malaria infection only in rural settings in 2015 (OR 0.88, 95% CI 0.83–0.93, p < 0.05), as well as in the effect of air-conditioning in the home, which was also found to significantly reduce the odds only in urban areas in 2015 (OR 0.73, 95% CI 0.58–0.92, p < 0.05) (Fig. 3; Additional file 1: Table S1).

### Environmental risk factors

Among environmental risk factors, most had non-significant effects across both urban and rural settings and between surveys. The exception was elevation, with each 100-m increase in elevation corresponding to around 20% lower the odds of malaria infection in people living in rural areas in 2015 (OR 0.78, 95% CI 0.68–0.90, p < 0.05) and 2018 (OR 0.79, 95% CI 0.58–1.06, p < 0.05) and around 50% lower in urban areas in 2015 (OR 0.48, 95% CI 0.35–0.66, p < 0.05) and 2018 (OR 0.55, 95% CI 0.40–0.75, p < 0.05). The confidence intervals for the other predictors (TCB, TCW, EVI, LST and slope) overlap with the null, precluding any strong inferences about changing risk of infection(Fig. 3; Additional file 1: Table S1).

## Discussion

**Fig. 4:**
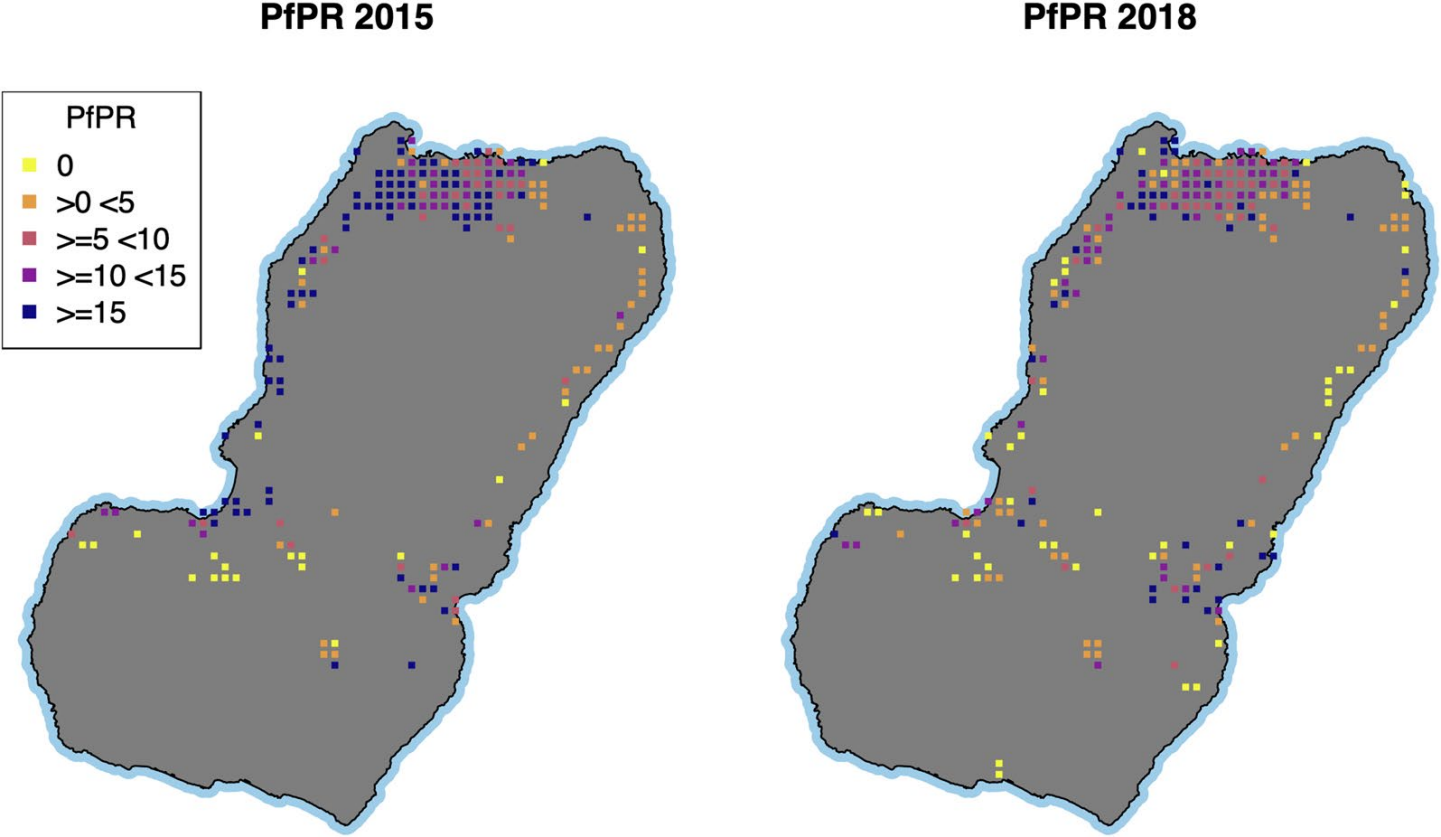
Malaria prevalence on Bioko Island in 2015 and 2018. Pixels represent 1 × 1 km inhabited areas. Raw *Pf*PR data from each Malaria Indicator Survey (MIS) were interpolated using kernel smoothing

This study investigated the relationship between malaria infection and individual, household, and environmental factors on Bioko Island. This investigation is important to better understand some of the drivers explaining the heterogeneity of malaria prevalence on the Island after many years of successful scaling-up of control interventions. Moreover, despite the intensive and continued efforts, reduction in prevalence on Bioko stalled since 2016, and the situation has worsened more recently in several areas across the Island (Fig. 4). Therefore, understanding some of the principal factors driving infection would prove critical to informing decision-making.

Several factors were investigated but, after adjusting for other covariates and demographic factors such as age and sex, which had a significant effect, only a handful were found to significantly influence the odds of malaria infection. History of travel to mainland EG proved by far the factor most strongly associated with increased odds of infection, with between a threefold and a fivefold increase in travellers. This finding was consistent across survey years and rural and urban areas, and is in agreement with previous investigations that have looked at the impact of malaria importation and its challenge for malaria control on Bioko Island [6, 7, 21, 27]. Human mobility patterns and malaria prevalence determined that travel to mainland EG explained much of the prevalence observed in and around Malabo, where travel prevalence is higher [7]. The fact that travel had a strong effect on the odds of infection in urban and rural communities and also remained consistent over time, despite a declining proportion of travellers in 2018 compared to 2015, points to a pervasive impact of this factor on the local malaria epidemiology. These findings underscore the need for new strategies to reduce the constant flow of parasites from the mainland to the Island, and these will probably be more cost-effective if focused on targeting high-risk frequent traveller groups [7, 25, 27, 28]. Further research to collect data and parameters from the mobile population could benefit in the understanding of this demographic and the development of tailored interventions. The studies can analyse their behaviours regarding access to case management in both locations, as well as their access to the use of bed nets and other vector control methods. In addition, MIS data might be utilized to explore the travel patterns of the mobile population [29], investigate the possible sources of acquired transmission during travel, and identify the risk of parasite importation in the locations where the mobile population goes. However, reducing transmission in mainland EG would be a critical long-term solution, though economically, logistically, and politically onerous.

The analyses also showed that bed net users had around 30% lower the odds of being infected with malaria, after adjusting for other covariates. This effect was similar in urban and rural areas. The BIMEP has deployed significant efforts to provide high coverage with LLINs through two triennial MDCs in 2015 and 2018. During these, households received a number of nets based on the household occupancy, the number of sleeping areas and the number of LLINs they already owned. In 2015 and 2018, 149,097 PermaNet 2.0 (deltamethrin, Vestergaard) and 155,972 Olyset Plus (permethrin plus the synergist, piperonyl butoxide (PBO), Sumitomo Chemical) were distributed, respectively. Following these MDCs, universal LLIN coverage was achieved based on the two critical indicators of household ownership and population access. Mean household ownership was 90.9% (IQR: 80.0–100%) and 92.0% (IQR: 82.7–100%) and mean population access 97.3% (IQR: 93.8–100%) and 82.9% (IQR: 73.9–100%) in 2015 and 2018, respectively. Soon after distribution, however, these indicators dropped significantly, as did LLIN use, from 53.6% (IQR: 36.2–67.9%) to 36.6% (IQR: 12.7–58.1%) and 52.2% (IQR: 33.3–68.3%) to 28.7% (IQR: 14.4–45.5%) a year after each MDC (unpublished data), suggesting that, unlike many other malaria endemic settings [30] the net access-use gap on Bioko is substantial, geographically heterogeneous, and driven by socio-demographic and behavioural factors. Despite net-use decreasing significantly soon after distribution, by using data from the MIS that took place soon after each MDC, we were able to get a reliable measure of the impact of LLIN use on reducing the risk of malaria infection, reinforcing the need to reimagining distribution and communication strategies that can improve adherence to LLINs by the population.

The effect of household-level factors on the odds of malaria infection showed a mixed picture, with confidence intervals often overlapping the null. Larger and overcrowded households were found to generally increase the odds of malaria infection after adjusting for SES, which is a likely confounder. This effect could be driven by low intrahousehold LLIN population access, but the timing of the MIS soon after each MDC again guaranteed that this indicator was still high among surveyed households. In other studies, it has been suggested that smaller density households have a lower risk because fewer people sleeping in the same room at night decreases the probability of transmission of the parasite to other hosts [31]. In nearby Cameroon, household overcrowding was identified as a major determinant of malaria mortality amongst infants [32]. A similar finding was observed for malaria prevalence amongst children in peri-urban areas of The Gambia, who had almost double the odds of malaria infection if they slept in rooms with more than three people [33]. Similarly, a study of the frequency of malaria events in children under five years old in a Sudanese village found that households with more than five people had more than double the odds of malaria infection [34]. People living in houses with a single sleeping room were also at higher risk of malaria in a highland area of Ethiopia compared to households with multiple sleeping rooms [35]. Intriguingly, the effect of household overcrowding on Bioko was not consistent across surveys and across rural and urban settings (Fig. 4). There are many households with low-occupancy on the Island, and targeting malaria control interventions to pre-identified high-risk households could prove a cost-effective malaria control strategy that needs to be further assessed. This would be logistically possible to implement using spatial decision support tools developed by the BIMEP [20, 36]. A potential caveat is that the Island’s population is highly mobile [7] and residents are often away during planned interventions in their communities [36], possibly making it difficult to target and getting access to specific households for interventions.

It was also identified that certain housing conditions (*i.e.,* open eaves, absence of air-conditioning) could increase the odds of malaria infection but this effect was identified only in urban areas and only in 2015. Previous analyses had shown associations between housing characteristics and malaria prevalence on Bioko [37], suggesting that improving basic housing standards could significantly reduce the odds of malaria infection, particularly in urban areas, where open eaves were a significant risk factor [38, 39]. Household vector control interventions have been motivated by the assumption that the primary anopheline vectors on Bioko Island are endophagic and bite human hosts within their dwellings. If this were the case, then improving housing characteristics could reduce the risk of malaria infection across different settings primarily by hampering house entry of vector mosquitoes [35, 38, 40]. In an endemic area of Sri Lanka, poor housing was linked to a 2.5-fold higher risk of malaria infection compared to residents living in houses of good construction [41]. The risk was further increased when housing was located near anopheline breeding sites. In Eswatini, adjusted models showed that people inhabiting medium and low-quality construction homes had one and a half and twice the odds of malaria infection than individuals living in high-quality constructions [39]. Open eaves and unscreened windows were also identified as significant risk factors in a hypoendemic malaria setting [42]. House screening interventions, for example, have been shown to reduce anopheline vector densities and anemia in children in homes in The Gambia [43, 44]. In Ethiopia, entomological inoculation rates, malaria prevalence, and incidence in houses intervened with window, and door screening were lower than in houses without screening [40]. Ceiling modifications reduced indoor vector densities by around 80% in houses in Western Kenya [45]. Considering the cost of LLINs and IRS, investing in improving housing conditions for the general population may prove a cost-effective strategy in the middle to long term [38, 41, 46] and requires further investigation in the context of Bioko Island [37]. This argument, however, is challenged by recent observations of increased outdoor biting activity of mosquito vectors on Bioko, which is subject of current investigation by the BIMEP (unpublished work). This changing mosquito behaviour could partly explain the lack of significant associations between housing characteristics and malaria infection over time and in urban and rural dwellings.

Finally, with the exception of elevation, the investigation of environmental covariates could not reveal any strong effects on the odds of malaria infection on Bioko. This could be explained by the generally homogenous climatic conditions across the Island, where very hot and humid conditions prevail for much of the year. This echoes previous observations of small correlation coefficients of environmental predictors in Bayesian geo-statistical models predicting malaria prevalence [7]. The effect of elevation is not surprising but, unfortunately, this effect is only observable in a few communities on Bioko, as the highest altitude regions correspond to two uninhabited nature reserves in the center of the Island.

In a changing landscape of malaria transmission on Bioko Island, there is an increasing need for adaptively managing malaria control interventions. Between 2015 and 2019, IRS was targeted to areas where prevalence was relatively high, leaning on triennial MDC as the principal vector control strategy to guarantee high LLIN coverage to the whole island population. Malaria prevalence has proven a moving target, however, and the poor uptake of bed nets by the population adds to considerable challenges. Moreover, recent changes in vector ecology point to the need to complement existing interventions with novel strategies that also tackle outdoor and earlier biting [47, 48]. Notwithstanding, knowledge of some of the most important risk factors at the individual and household level could help redefine the optimal suite of malaria control interventions [49]. The effect of housing characteristics on the risk of malaria infection is worth considering for targeting certain household interventions using existing tools [36]. Crucially, the analyses presented here confirmed the very significant contribution of travel to the risk of being infected with malaria parasites, and this represents yet another call for interventions that target malaria importation and travellers. These could take the form of chemoprophylaxis or test-and-treat strategies for travellers at points of entry or, in perhaps a not-too-distant future, travel vaccines. Based on the results presented here and in previous work [27] it is plausible that this approach could substantially impact the local malaria burden.

## Conclusion

Malaria risk on Bioko Island is heterogeneous and determined by a combination of factors interacting with local mosquito ecology. These interactions warrant further investigation to better adapt control according to need. The single most important risk factor identified was travel to mEG, in line with previous studies, and represents a significant challenge for the success of malaria control on the Island. Strategies to continue reducing the burden of transmission on the island begin with control activities on the mainland, a better understanding of the migratory population to tackle malaria among this group with targeted interventions, and a focus on importation upon return to the island. For Equatorial Guinea to achieve its goal of eliminating malaria at a national and sub-national level, there must be a focus on malaria prevention among the mobile population.

## Supplementary Information

**Additional file 1: Table S1.** Odds ratios estimated from regression models of all risk factors for malaria transmission on Bioko Island and 95% confidence intervals, stratified by rural and urban settings and malaria annual indicator survey year, 2015 and 2018.

## Data Availability

The datasets used and/or analysed during the current study are available from the corresponding author upon reasonable request.
